# *E*-cigarette use among adolescents in Latin America: A systematic review of prevalence and associated factors

**DOI:** 10.1016/j.pmedr.2024.102952

**Published:** 2024-12-19

**Authors:** Juan S. Izquierdo-Condoy, Kenny Ruiz Sosa, Camila Salazar-Santoliva, Natalia Restrepo, Guillermo Olaya-Villareal, Juan S. Castillo-Concha, Valentina Loaiza-Guevara, Esteban Ortiz-Prado

**Affiliations:** a One Health Research Group, Faculty of Medicine, Universidad de las Américas, Quito, Ecuador; b Facultad de Medicina, Fundación Universitaria Autónoma de las Américas, Pereira, Colombia; c Area de Medicina Interna, Universidad de Cartagena, Cartagena, Colombia; d Facultad de Ciencias de la Salud, Universidad del Quindío, Armenia, Colombia

**Keywords:** *E*-cigarettes, Adolescents, Use, Associated factors, Latin America

## Abstract

**Background:**

Electronic cigarettes, introduced as a safer tobacco alternative, have unintentionally exposed millions of youths to nicotine and harmful chemicals. Adolescence, a key period for forming lifelong habits, has seen rising e-cigarette use, particularly in developing regions like Latin America, warranting thorough investigation.

**Objective:**

To describe the prevalence and factors associated with e-cigarette use among adolescents in Latin America.

**Methods:**

A systematic review was conducted according to PRISMA guidelines, covering studies published between 2003 and May 2024. Database searches included PubMed/Medline, Web of Science, Scopus, Google Scholar, Scielo, and LILACS. A total of 582 studies were identified, of which 14 met the inclusion criteria. Study data were synthesized and assessed using the Newcastle-Ottawa Scale and Joanna Briggs Institute checklist.

**Results:**

The prevalence of e-cigarette among adolescents in six Latin American countries ranged from 2.6 % to 64.2 %, with a pooled mean prevalence of 18.9 %, and lifetime use higher than current use. Key associated factors included male sex, concurrent use of tobacco and other substances, social influences, and exposure to online advertising. A widespread lack of knowledge regarding e-cigarette risks, coupled with limited regulatory oversight, may be contributing to higher usage rates.

**Conclusion:**

This review underscores critical gaps in data on adolescent e-cigarette use in Latin America and highlights the need for expanded research and targeted public health interventions. Nearly one-fifth of adolescents reported using e-cigarettes. Comprehensive prevention programs addressing factors associated with adolescent e-cigarette use in Latin America, involving diverse stakeholders and integrating education, school-based policies, social media campaigns, and policy restrictions, are strongly recommended.

## Highlights

• E-cigarette use among Latin American adolescents ranges from 2.4 % to 64.2 %, with a pooled mean prevalence of 18.9 %.
• Tobacco, substance use, and online ads are key factors linked to e-cigarette use in adolescents.
• Knowledge gaps exist: 6 %–86 % of adolescents are unaware of e-cigarette risks.
• Limited research exists in key countries, such as Brazil, Peru, Chile, and Uruguay.
• Targeted prevention programs and stricter regulations are urgently needed.

## 1 Introduction

Electronic cigarettes vaporize a liquid, often with additives such as flavors and nicotine, creating an aerosol. They are classified as nicotine delivery systems (ENDS) or nicotine-free systems (SESN) (Sala and Gotti, 2023). Initially marketed as an alternative to traditional tobacco, e-cigarettes present significant health risks, including increased cardiovascular and lung disease, exposure to toxins, risk of physical injury, and potential nicotine dependence (Hamann et al., 2023; Izquierdo-Condoy et al., 2024a; Rose et al., 2023). Moreover, while these devices have been shown to be a potential tool in reducing tobacco smoking especially among smokers with high nicotine dependence (Selya et al., 2018), they have also shown side effects by being able to trigger nicotine dependence even in adolescents (Case et al., 2018; Sreeramareddy et al., 2023), to which could be added a possible “gateway” effect reflected by a slowdown in cessation of tobacco cigarette use since the advent of e-cigarettes (Harrell et al., 2022).

Given the rapid distribution of these devices over the last decade, reports show that their use among adolescents is becoming more common (Dautzenberg et al., 2023). The World Health Organization (WHO) has established that by 2023, the consumption of electronic cigarettes will have higher rates among adolescents aged 13 to 15 years than among adults (World Health Organization, 2023). Examples presented by the WHO include Canada and the United Kingdom, where e-cigarette use among adolescents doubled and tripled, respectively, between 2017 and 2022 (World Health Organization, 2023). In Europe, 32 % of 15-year-olds have tried electronic cigarettes, compared to 15 % who have used conventional tobacco (World Health Organization, 2024). Similarly, estimates by the Pan American Health Organization (PAHO) suggest that by 2024, 5.4 % of adolescents in the Americas would use e-cigarettes, approaching the 6 % who consume conventional cigarettes (Pan American Health Organization, 2024).

*E*-cigarette regulations remain unclear in several regions. In the Americas, in countries like El Salvador, and Ecuador existing tobacco regulations have been extended to include e-cigarettes with minimum age restrictions and indoor use bans, yet insufficient sales oversight enables easy access for adolescents (Institute for Global Tobacco Control, 2020). Conversely, countries like Brazil, Venezuela, Argentina, Uruguay, Nicaragua, Panama, Suriname and Mexico have implemented comprehensive bans on the manufacture, sale, and possession of these devices (Pan American Health Organization, 2023).

Adolescence is a critical stage of life where behaviors and habits are developed and defined, often lasting into adulthood (Jaworska and MacQueen, 2015), with smoking frequently beginning at this stage (Grapatsas et al., 2017; Pan American Health Organization, 2024; Park, 2011). In recent years, alongside the increase in tobacco consumption among adolescents, a new trend has emerged: the availability of innovative alternatives to tobacco, such as electronic cigarettes, which adolescents can access more easily and perceive positively (Aly et al., 2022; Izquierdo-Condoy et al., 2024b; Kim et al., 2019).

Despite their growing popularity, research on e-cigarette use among adolescents is limited in developing regions, including Latin America. This lack of knowledge is even more pronounced among teenagers. Given the cultural, socioeconomic, and legislative differences in the region, there is a need to better understand e-cigarette use among adolescents. Therefore, the objective of this research is to systematically describe the prevalence and factors associated with the use of electronic cigarettes among adolescents in Latin America.

## 2 Materials and methods

### 2.1 Study design

We conducted a systematic review based on the Preferred Reporting Items for Systematic Reviews and Meta-Analyses (PRISMA) methodological guidelines. This research was based on the “PEO” and “CoCoPop” methodological guides for systematic reviews in health sciences (Munn et al., 2018), establishing the strategy:

• Condition: epidemiology of electronic cigarette use
• Context: Latin America
• Population: teenagers
• Exposure: electronic cigarette use
• Result: prevalence and factors associated with the electronic cigarette use

### 2.2 Search strategies

We conducted an in-depth bibliographic search in English and Spanish languages  to cover the broadest scope of academic literature. We use several key databases and libraries for Latin American scientific literature, including PubMed/Medline, Web of Science, Scopus, Google Scholar, Scielo, and Latin American and Caribbean Literature in Health Sciences (LILACS). Additionally, we employed a snowball strategy to review reference lists of relevant articles for overlooked studies. Our bibliographic search focused on primary studies published between 2003 (the time when the purchase of electronic cigarettes began) (van der Eijk et al., 2021) and May 2024.

To run the search, we used the syntax of the following index terms, keywords, and Boolean operators: (“Electronic Nicotine Delivery Systems” OR “Electronic Cigarettes” OR “Electronic Cigarettes” OR “Vaping” OR “Nicotine Vaping” OR “Vape”) AND (“use” OR “usage” OR “consume”) AND (“prevalence” OR “frequence”) AND (“adolescents” OR “youth”) AND (“Latin America” OR “Belize” OR “Costa Rica” OR “Cuba” OR “El Salvador” OR “Guatemala” OR “Honduras” OR “Nicaragua” OR “Panama” OR “Mexico” OR “Puerto Rico” OR “Colombia” OR “Venezuela” OR “Ecuador” OR “Brazil” OR “Bolivia” OR “Chile” OR “Argentina” OR “Uruguay” OR “Paraguay” OR “Peru” OR “Haiti”) in the title (TI) or abstract (AB).

### 2.3 Selection criteria

#### 2.3.1 Inclusion criteria

Studies in English or Spanish. Primary studies that examine the prevalence and factors associated with the use or consumption of electronic cigarettes among adolescents in Latin America.

**2.3.2 Exclusion criteria.**

- Studies that investigate the use and factors associated with the consumption of substances other than electronic cigarettes, such as tobacco or marijuana, in adolescents in Latin America.

- Secondary research on the use and factors associated with the use of electronic cigarettes in adolescents in Latin America such as systematic reviews, narrative reviews, scoping reviews and meta-analysis.

- Non-original research-type papers on the use and factors associated with the use of electronic cigarettes in adolescents in Latin America such as conference articles, perspectives, comments, opinion articles and editorial letters.

### 2.4 Selection of studies

**Fig. 1:**
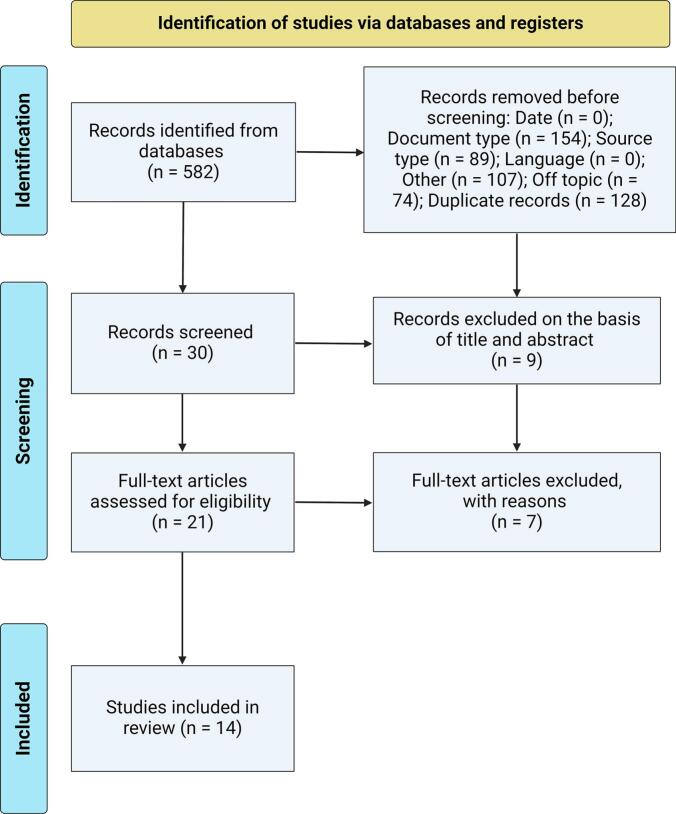
PRISMA flowchart illustrating the study selection process for this systematic review, detailing the number of studies screened, assessed for eligibility, and included in the review, with reasons for exclusions at each stage.

The bibliographic search initially yielded a total of 582 articles. In the first screening phase, 552 studies were eliminated, mainly due to document type that included different types of non-original research manuscripts (*n* = 154), and 128 studies were eliminated due to duplicates. Of the remaining 30 articles, 9 studies were excluded due to limitations found in the title or abstract. Finally, the 21 eligible articles were reviewed in their entirety and 14 studies were included in this research (Fig. 1).

### 2.5 Bias assessment

To minimize the risk of bias, two members of the research team (JSIC, KRS) independently performed the data extraction process at different times. Any discrepancies found during the data collection phase were resolved through discussion until consensus was reached among all members of the research team. This method was implemented to ensure the accuracy and reliability of our findings.

### 2.6 Critical evaluation and data synthesis

A comprehensive review was conducted of all manuscripts that met the established selection criteria. For cohort studies, we used the Newcastle-Ottawa Quality Assessment Scale for quantitative analysis; developed to evaluate the quality of non-randomized studies with its design, content and ease of use aimed at the task of incorporating quality assessments in the interpretation of meta-analysis results (Wells et al., 2021). For cross-sectional studies, the Joanna Briggs Institute (JBI) critical appraisal checklist for analytical cross-sectional studies (Joanna Briggs Institute, 2024) was used.

Information from these manuscripts was extracted, meticulously organized, and synthesized into descriptive tables. This format was chosen to present our results in a clear and concise manner, facilitating reader understanding. Additionally, values related to adolescent e-cigarette use prevalence from the included studies were extracted and pooled to calculate the mean prevalence and standard deviation of e-cigarette use in the region.

## 3 Results

A total of 14 studies were included in this review. Regarding quality assessment, it was found that the four longitudinal studies (Barrientos-Gutierrez et al., 2019; Morello et al., 2018, Morello et al., 2016; Thrasher et al., 2016) showed good quality (at least 6 out of 9, according to the Newcastle-Ottawa scale) (Supplementary Table 1). Among the ten cross-sectional studies using the JBL critical appraisal scale, quality was variable, with four studies of high quality (Chérrez-Ojeda et al., 2024; Gottschlich et al., 2020; Rodríguez-Bolaños et al., 2020; Zavala-Arciniega et al., 2019), 3 studies of moderate quality (Barrera-Núñez et al., 2023; Cortés Pérez et al., 2023; Morello et al., 2020) and 3 studies of low quality (Isea López et al., 2023; Ocasio-Peña et al., 2023; Scoppetta et al., 2021) (Supplementary Table 2).

The majority of the research (*n* = 8) was carried out in Mexico, followed by Argentina (*n* = 3), with studies published between 2016 and 2024. The most common research design was cross-sectional (*n* = 10), mostly involving large adolescent populations (12 studies with more than 1000 individuals) (Table 1).

**Table 1:** Systematic summary of studies evaluating the prevalence of use and factors associated with the use of e-cigarettes among adolescents in Latin America, based on research published between 2016 and 2024 (systematic review conducted in 2024).

Author	Country (Region/city)	Study design	Population	EC prevalence of use	Associated Factors	Knowledge and beliefs	Patterns of EC use
Ocasio-Peña C., et al., 2023 (Ocasio-Peña et al., 2023)	Puerto Rico	Cross sectional	Puerto Rican adolescents (*n* = 8603)	21.9 %	N/A	22.1 % do not know the components of EC.	N/A
Barrera-Núñez D., et al., 2023 (Barrera-Núñez et al., 2023)	Mexico	Cross sectional	Mexican adolescents from the Continuous National Health and Nutrition Survey (*n* = 19,230,769 approximately)	2.6 %	N/A	N/A	N/A
Cortés E., et al., 2023 (Cortés Pérez et al., 2023)	Mexico (Guanajuato)	Cross sectional	Mexican adolescents (*n* = 355)	64.2 %	N/A	91.3 % know risks of EC use.	N/A
Zavala-Arciniega L., et al., 2020 (Zavala-Arciniega et al., 2019)	Mexico (Mexico City, Guadalajara, and Monterrey)	Cross sectional	Adolescents of last year of middle school (*n* = 8718)	20.5 %	Being men, regular tobacco use, drug use in the last year, seeking sensation.	70.0 % use EC for curiosity.	55 % use liquid with nicotine, 46 % prefer fruit flavors
Rodríguez-Bolaños R., et al., 2020 (Rodríguez-Bolaños et al., 2020)	Mexico (Mexico City, Guadalajara, and Monterrey)	Cross sectional	Adolescents of public middle school (*n* = 4576)	8.0 %	Drinking, technophilia, having friends who smoke.	N/A	N/A
Gottschlich A., et al., 2020 (Gottschlich et al., 2020)	Guatemala (Guatemala City)	Cross sectional	Adolescents of private school (*n* = 2870)	11.3 % (8.4 former users; 2.9 % current users)	Current tobacco use, moderate alcohol consumption, marijuana use in past 30 days, having friends who use EC.	N/A	Start of consumption at 15.09 years old
Morello P., et al., 2018 (Morello et al., 2018)	Mexico	Longitudinal follow-up	High school adolescents (*n* = 4877)	9.6 %	N/A	N/A	N/A
Barrientos-Gutierrez I., et al., 2019 (Barrientos-Gutierrez et al., 2019)	Mexico (Mexico City, Guadalajara, and Monterrey)	Longitudinal follow-up	Adolescents of public middle school (*n* = 8123)	31.0 % (19.0 % former users; 12.0 % current users)	Higher technophilia, being male, alcohol tried, drugs tried, exposure to EC online ads, tobacco smoking	N/A	N/A
Thrasher J., et al., 2016 (Thrasher et al., 2016)	Mexico (Mexico City, Guadalajara, and Monterrey)	Longitudinal follow-up	First year students of public middle schools (*n* = 10,146)	10 %	Being male, having parents or friends who use tobacco, higher sensation seeking, alcohol and drug use, and perceiving EC are less harmful than tobacco.	49 % do not know risks of EC, 18 % perceive less risk than tobacco smoke	40 % prefer fruit flavor
Morello P., et al., 2016 (Morello et al., 2016)	Argentina (Buenos Aires, Cordoba, Tucumán)	Longitudinal follow-up	Public and private schools' secondary students (*n* = 3172)	7.6 %	Tobacco use, alcohol consume, drugs ever use, sensation seeking, having tobacco users as friends, attending to private schools, and higher exposure to ads for tobacco products	6 % perceive less risk than tobacco smoke, 86 % unaware EC use	N/A
Chérrez-Ojeda I., et al., 2024 (Chérrez-Ojeda et al., 2024)	Ecuador	Cross sectional	Students from 10 years and older (*n* = 1854 from 10 to 17 years)	21.8 % (14.1 % former users; 7.7 % current users)	N/A	N/A	Start of consumption at 13.9 years old
Isea S., et al., 2023 (Isea López et al., 2023)	Venezuela (Caracas)	Cross sectional	High school adolescents (n = N/A)	N/A	Alcohol use	N/A	N/A
Scoppetta O., and Villamil A., 2023 (Scoppetta et al., 2021)	Colombia (Bogota)	Cross sectional	High school adolescents (*n* = 1707)	32.3 % ever use; 15.8 % current use	N/A	Flavors and curiosity are the main reasons to first use. 43.4 % believe EC is less harmful than tobacco.	Start of consumption at 13.6 years old
Morello P., et al. 2020 (Morello et al., 2020)	Argentina	Cross sectional	Argentinian adolescents from 13 to 15 years old (*n* = 1251)	21.5 % (14.4 % former users; 7.1 % current users)	Tobacco use.	17.8 % possibly use EC in the next 12 months. 42 % believe EC is less harmful than tobacco. 40 % do not know the harmful effects of EC.	N/A
Morello P., et al., 2018 (Morello et al., 2018)	Argentina	Longitudinal follow-up	High school adolescents (*n* = 1680)	2.4 %	N/A	N/A	N/A

EC: electronic cigarette, N/A: not available information. Some of the studies included in the table may have evaluated several populations of adolescents from more than one country, in which case the same study may be mentioned more than once.

### 3.1 Prevalence of electronic cigarette use

**Fig. 2:**
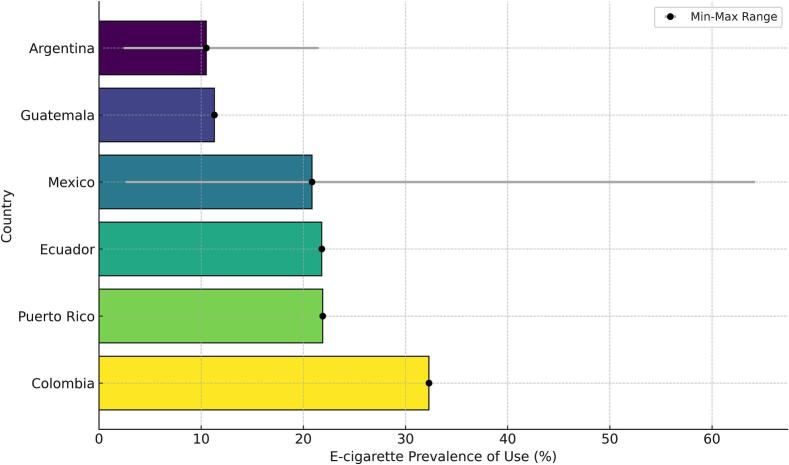
Prevalence of e-cigarette use among adolescents in six Latin American countries, based on studies published between 2016 and 2024 (systematic review conducted in 2024). Error bars indicate the minimum and maximum prevalence reported in studies for each country.

A total of 13 studies reported prevalence data on e-cigarette use among adolescents from various Latin American countries. The study by Isea S. et al. in Venezuela was the only one that did not provide prevalence estimates. For the other studies, which included data from Puerto Rico, Mexico, Guatemala, Colombia, Ecuador, and Argentina, the prevalence of e-cigarette use ranged widely, from 2.4 % in a sample of 1680 high school students in Mexico and Argentina (Morello et al., 2018), to 64.2 % among 355 adolescents in Mexico (Cortés Pérez et al., 2023). In addition, the combined analysis across these six countries yielded a pooled mean prevalence of 18.9 % (±15.6 %) among Latin American adolescents (Barrera-Núñez et al., 2023; Barrientos-Gutierrez et al., 2019; Chérrez-Ojeda et al., 2024; Cortés Pérez et al., 2023; Gottschlich et al., 2020; Morello et al., 2020, Morello et al., 2018, Morello et al., 2016; Ocasio-Peña et al., 2023; Rodríguez-Bolaños et al., 2020; Scoppetta et al., 2021; Thrasher et al., 2016; Zavala-Arciniega et al., 2019) (Table 1 and Fig. 2).

Additionally, five studies included distinctions between past and current e-cigarette use among adolescents from Guatemala, Mexico, Ecuador, Colombia, and Argentina. Past e-cigarette use ranged from 8.4 % in Guatemala to 19.0 % in Mexico. For current e-cigarette use, prevalence estimates ranged from 2.9 % in Guatemala to 15.8 % among Colombian adolescents (Barrientos-Gutierrez et al., 2019; Chérrez-Ojeda et al., 2024; Gottschlich et al., 2020; Morello et al., 2020; Scoppetta et al., 2021) (Table 1).

### 3.2 Factors associated with electronic cigarette use

Eight studies identified various factors associated with e-cigarette use among adolescents in Latin America. Individual factors included sex, with males showing a higher likelihood of use (APR: 2.46, 95 %CI 1.75–3.47), and technophilia, which was positively associated with e-cigarette use (ARR: 1.27, 95 %CI 1.05–1.55; AOR: 1.17, 95 %CI 1.08–1.28), particularly at higher levels (APR: 1.84, 95 %CI 1.06–3.09). Substance use behaviors showed significant associations, with adolescents who had a history of tobacco use displaying higher association with e-cigarette use (AOR: 3.73, 95 %CI 2.03–6.82; AOR: 1.79, 95 %CI 1.48–2.17; AOR: 2.50, 95 %CI 2.02–3.11) and those with current tobacco use demonstrating markedly elevated association (AOR: 46.12, 95 %CI 16.15–131.74; AOR: 4.65, 95 %CI 3.7–5.84; AOR: 2.58, 95 %CI 1.38–4.83; AOR: 3.13, 95 %CI 2.40–4.08; APR: 1.81, 95 %CI 1.03–3.19). Additionally, drug use in the past year (APR: 1.89, 95 %CI 1.25–2.86), current alcohol consumption (AOR: 2.53, 95 %CI 1.08–5.95; COR: 2.57, 95 %CI 1.71–3.84; ARR: 1.85, 95 %CI 1.09–3.15), and ever alcohol consumption (AOR: 1.30, 95 %CI 1.06–1.61; AOR: 1.34, 95 %CI 1.13–1.60) were associated with increased likelihood of e-cigarette use. Other associated factors included marijuana use in the last 30 days (AOR: 2.29, 95 %CI 1.27–4.13), lifetime drug use (COR: 4.66, 95 %CI 2.44–8.90; AOR: 2.03, 95 %CI 1.68–2.46), and sensation-seeking behavior (AOR: 1.13, 95 %CI 1.05–1.14; AOR: 1.46, 95 %CI 1.18–1.81; AOR: 1.14, 95 %CI 1.05–1.24; APR: 1.31, 95 %CI 1.02–1.68) (Table 1).

Environmental influences were also significant, with factors such as having family members who smoke (AOR: 1.7, 95 %CI 1.19–2.45; AOR: 1.52, 95 %CI 1.24–1.85; AOR: 1.47, 95 %CI 1.23–1.76), or friends who smoke (AOR: 1.52, 95 %CI 1.17–1.99; AOR: 1.82, 95 %CI 1.25–2.99; ARR: 1.44, 95 %CI 1.02–2.01; AOR: 1.37, 95 %CI 1.16–1.61) correlating with higher e-cigarette use. Peer influence played a role, as having friends who used e-cigarettes increased odds substantially (AOR: 5.03, 95 %CI 3.73–6.78). Exposure to e-cigarette advertisements online (AOR: 2.95, 95 %CI 1.97–3.39; AOR: 1.87, 95 %CI 1.04–3.36), and frequent exposure to tobacco ads (AOR: 1.45, 95 %CI 1.12–1.86), were also linked to e-cigarette use, as was the perception that e-cigarettes are less harmful than conventional tobacco (AOR: 3.27, 95 %CI 2.66–4.01) (Table 1). These findings derive from studies on Mexican, Guatemalan, Argentine, and Venezuelan adolescents (Barrientos-Gutierrez et al., 2019; Gottschlich et al., 2020; Isea López et al., 2023; Morello et al., 2020, Morello et al., 2016; Rodríguez-Bolaños et al., 2020; Thrasher et al., 2016; Zavala-Arciniega et al., 2019).

### 3.3 Knowledge and beliefs about electronic cigarette use

Discrepancies were identified regarding knowledge of the devices. While the study by Cortés E. et al. among Mexican adolescents (*n* = 355) showed that 91.3 % knew the risks of using electronic cigarettes (Cortés Pérez et al., 2023), the longitudinal study by Morello P. et al. among public and private high school students in Argentina revealed that 86 % and 49 % of Mexican adolescents were unaware of the risks of electronic cigarettes (Morello et al., 2016; Thrasher et al., 2016), and 22.1 % of Puerto Ricans were unaware of the components of electronic cigarettes (Ocasio-Peña et al., 2023). Furthermore, data from Mexican, Argentine, and Colombian adolescents showed that between 6 % and 43.4 % believe that electronic cigarettes have fewer risks than tobacco cigarettes (Morello et al., 2020, Morello et al., 2016; Scoppetta et al., 2021; Thrasher et al., 2016). Curiosity was the main reason for use among Mexican adolescents, while among Colombians, it ranged between 18 % and 70 % (Cortés Pérez et al., 2023; Scoppetta et al., 2021; Zavala-Arciniega et al., 2019). Flavors and taste for electronic cigarettes were also mentioned (Scoppetta et al., 2021; Zavala-Arciniega et al., 2019). Regarding future intentions, 84.6 % of Mexican adolescents plan to quit electronic cigarettes (Cortés Pérez et al., 2023), whereas 17.8 % of Argentine adolescents (aged 13 to 15) consider continuing to use them in the next 12 months (Morello et al., 2016). The average age of onset ranged between 13.6, 13.9, and 15.09 years among Colombian, Ecuadorian, and Guatemalan adolescents (Chérrez-Ojeda et al., 2024; Gottschlich et al., 2020; Scoppetta et al., 2021).

## 4 Discussion

In this systematic review of 14 studies, we investigated the prevalence and associated factors of e-cigarette use among adolescents in Latin America. Among the 13 studies reporting prevalence data, rates of e-cigarette use ranged from 2.6 % to 64.2 %, with an overall mean prevalence of 18.9 % across six countries (Barrera-Núñez et al., 2023; Barrientos-Gutierrez et al., 2019; Chérrez-Ojeda et al., 2024; Cortés Pérez et al., 2023; Gottschlich et al., 2020; Isea López et al., 2023; Morello et al., 2020, Morello et al., 2018, Morello et al., 2016; Ocasio-Peña et al., 2023; Rodríguez-Bolaños et al., 2020; Scoppetta et al., 2021; Thrasher et al., 2016; Zavala-Arciniega et al., 2019). Furthermore, among the five studies that distinguished between current and past use, we found that the prevalence of current use (ranging from 2.9 % to 15.8 %) was lower than that of past use (ranging from 8.4 % to 19.0 %) among adolescents (Barrientos-Gutierrez et al., 2019; Chérrez-Ojeda et al., 2024; Gottschlich et al., 2020; Morello et al., 2020; Scoppetta et al., 2021). We attribute the wide variability in prevalence estimates to differences in study design, sample size, data collection methodologies, and timeframes (2016–2024). Notably, the prevalence identified for the region in this review is higher than that reported by Ling et al. in a systematic review of Southeast Asian adolescents, where rates ranged from 3.3 % to 11.8 % (Jane Ling et al., 2023). It also closely approximates but exceeds the international prevalence estimate by Kim et al., who reported 15.3 % among young populations in 2022 (Kim et al., 2022), indicating a likely recent increase in e-cigarette use among Latin American youth. The prevalence rates identified in this review may also be influenced by differences in participant age groups within the included studies, as most focused on specific age subgroups, such as adolescents aged 13 to 15 or secondary school students, which may affect the representativeness of the results.

Surprisingly, studies on e-cigarette use among adolescents were identified in only seven Latin American countries (Guatemala, Puerto Rico, Mexico, Argentina, Colombia, Ecuador, and Venezuela), underscoring the limited research and information available on this phenomenon in the region. Notably, despite substantial scientific contributions from countries such as Brazil and Peru, no studies were available from these populations. Furthermore, the data strongly support the need for expanded research on the prevalence of e-cigarette use and its associated factors in additional Latin American countries, including Chile, Uruguay, and Cuba, which have some of the highest tobacco use rates in the region (World Health Organization, 2022). given the lack of e-cigarette regulation in most Latin American countries, which makes these devices readily accessible to various demographic groups, including adolescents (Izquierdo-Condoy and Ortiz-Prado, 2024). Future research should prioritize these underrepresented nations to gain a broader understanding of e-cigarette use and to guide regulatory and public health interventions tailored to each country's specific needs. Expanding the geographical scope of research would yield a more comprehensive view of the prevalence and the social, cultural, and policy-related factors influencing e-cigarette use across Latin America.

The absence of regulatory frameworks for these devices, combined with the persistently high prevalence of tobacco cigarette use (38.5 %) in Latin America (Urrutia-Pereira et al., 2019), is concerning. Our review identified a strong association between tobacco and e-cigarette use among adolescents, with tobacco use emerging as the most common factor associated with e-cigarette use in five studies, all conducted in Mexico and Argentina (Morello et al., 2020, Morello et al., 2016; Rodríguez-Bolaños et al., 2020; Thrasher et al., 2016; Zavala-Arciniega et al., 2019). Additionally, findings indicate that having friends or family who smoke tobacco cigarettes is also linked to e-cigarette use among adolescents (World Health Organization, 2022), which further predisposes youth in the region to higher e-cigarette use.

Regarding associated factors, this review identified that being male is associated with e-cigarette use among adolescents, similar to findings among adolescents in the United States (Krishnan-Sarin et al., 2015), Southeast Asia, and Australia (Leung et al., 2023). Technophilia, defined as a positive orientation toward the use of new technologies, was also shown to be an important factor associated with e-cigarette use; however, this association has only been observed in adolescents from Mexico (Barrientos-Gutierrez et al., 2019; Rodríguez-Bolaños et al., 2020). We believe that this association may be closely related to the availability of online advertisements for e-cigarettes, as identified in 2 studies of Mexican and Argentine adolescents (Barrientos-Gutierrez et al., 2019; Morello et al., 2016), similar to findings by Amin S. et al. in Australian adolescents (Amin et al., 2020). In the Latin American context, this may be due to the relatively unregulated sale of these products online, which has been shown to increase the number of purchases by minors in the United States (Williams et al., 2015).

Furthermore, the concomitant use of other substances, including tobacco, drugs, marijuana, and alcohol, was identified as the most frequent factor associated with e-cigarette use among adolescents in Mexico, Argentina, and Venezuela (Barrientos-Gutierrez et al., 2019; Gottschlich et al., 2020; Isea López et al., 2023; Morello et al., 2020, Morello et al., 2016; Thrasher et al., 2016; Zavala-Arciniega et al., 2019). These findings are consistent with studies from around the world, including the United States, Hawaii, Hong Kong, Korea, and Thailand (Assanangkornchai et al., 2018; Demissie et al., 2017; Han and Chung, 2021; Jiang et al., 2016; Lozano et al., 2021; Park et al., 2020; Rothrock et al., 2020; Shih et al., 2017; Wills et al., 2015).

Knowledge about e-cigarettes among adolescents in Latin America shows a trend toward lack of knowledge. Despite being explored in only 6 studies, it was shown, for example, that in Puerto Rico, 22.1 % of adolescents do not know the components of e-cigarettes (Ocasio-Peña et al., 2023), while among Argentine adolescents, it was 40 % (Morello et al., 2020). Furthermore, among Mexican and Argentine adolescents, 6.9 % to 86 % are unaware of the risks of e-cigarette use (Cortés Pérez et al., 2023; Morello et al., 2016; Thrasher et al., 2016). Additionally, 6 % to 43.4 % of Mexican, Colombian, and Argentine adolescents believe that e-cigarettes pose less risk than tobacco cigarettes (Morello et al., 2020, Morello et al., 2016; Scoppetta et al., 2021; Thrasher et al., 2016). In Argentina, this belief has increased significantly, from 6 % among 3172 high school students in 2016 (Morello et al., 2016) to 42 % among 1252 adolescents aged 13 to 15 in 2020 (Morello et al., 2020). These findings align with research on adolescents from several other countries, including Thailand (Patanavanich et al., 2021), Hong Kong (Jiang et al., 2016), and Saudi Arabia (Doumi et al., 2023), where knowledge about e-cigarettes is also low.

This review appears to be the first systematic review exploring the issue of e-cigarette use among adolescents in Latin America. Despite exposing important characteristics related to e-cigarette use, aligned with the MPOWER strategies of the WHO Framework Convention on Tobacco Control (FCTC) (World Health Organization, 2024). Despite varying prevalence rates across the region, substantial e-cigarette use has been documented, driven by industry tactics targeting youth, appealing flavors, and high nicotine content, all of which increase the risk of addiction (Harrell et al., 2022; Izquierdo-Condoy and Ortiz-Prado, 2024). These findings underscore the urgent need for high-quality prevention programs tailored specifically for adolescents in Latin America.

Such programs should prioritize addressing the primary factors associated with adolescent e-cigarette use, including tobacco use and other substance use (e.g., alcohol and drugs) on both personal and social levels, especially given the influence of family and peer networks. Effective prevention strategies must incorporate health education and focus on new and emerging products, such as disposable vaporizers and youth-appealing flavors. To achieve meaningful impact, prevention efforts in Latin America should engage decision-makers and include schools, teachers, parents, and the broader community (Liu et al., 2022).

We suggest implementing interventions with a proven record in reducing youth tobacco use, such as school-based programs that use student leaders to influence peers' attitudes toward e-cigarette risks. These could be combined with smoke-free school policies and family-centered strategies, such as parental monitoring through educational programs that encourage parental involvement in risk communication around e-cigarettes (Duncan et al., 2018; Mylocopos et al., 2024)). Mass media campaigns in Latin America could also focus on emphasizing health risks and countering misconceptions about e-cigarette safety. Given the significant influence of technophilia and online e-cigarette advertising on adolescents, these campaigns should extend to social media platforms and include school-based programs designed to increase awareness of e-cigarette harms and reduce adolescents' likelihood of trying these devices, following successful examples in regions like the United States (Gaiha et al., 2021; Kelder et al., 2020).

Strengthening public policies around e-cigarette regulation is also crucial. Examples from other regions, such as requiring medical prescriptions for nicotine products, can limit access and focus these devices on adults seeking to quit tobacco use (Greenhalgh et al., 2022; Therapeutic Goods Administration (TGA), 2022). Specific bans on selling e-cigarettes to minors—both online and in person—are essential, though online enforcement poses challenges. Restrictions on sales of flavored e-cigarettes (e.g., fruit and candy flavors popular among adolescents) are also recommended (FDA- Office of the Commissioner, 2020; Greenhalgh et al., 2022). Additionally, as seen in this review and in prior studies, exposure to online advertising is strongly associated with adolescent e-cigarette use (Pettigrew et al., 2023). Thus, introducing public policies to restrict such advertising, especially online, could yield positive outcomes for Latin American youth.

### 4.1 Limitations

This systematic review has several limitations. The studies included come from a limited number of Latin American countries, with a significant concentration of research conducted in Mexico and Argentina, which limits the generalizability of the findings to the entire region. Additionally, the exclusion of articles published in Portuguese, the primary language of Brazil, may have led to the omission of literature from this significant Latin American population, thereby limiting the comprehensiveness of the review.

The heterogeneity of the studies in terms of design, sample size, and data collection methods presents challenges in comparing and synthesizing the results. Additionally, the variability in quality, particularly among the cross-sectional studies assessed by the JBL critical appraisal scale, further complicates the interpretation of the findings.

The age range of participants varied across studies, with some specifically focusing on younger adolescents (13–15 years) and others including older age groups. This variation may affect the representativeness and comparability of prevalence rates and associated factors. Moreover, the reliance on self-reported data in most studies introduces potential biases, such as recall bias and social desirability bias, which may influence the accuracy of reported e-cigarette use and associated behaviors.

## 5 Conclusions

Data on adolescent e-cigarette use in Latin America is limited, with several countries notably underrepresented, highlighting critical research gaps that demand further study. Reported prevalence varies widely, with pooled mean prevalence of 18.9 %, and current use generally lower than lifetime use.

Among adolescents who use e-cigarettes, associated factors include concurrent use of tobacco, alcohol, marijuana, and other substances, along with social influences and exposure to online advertising. These findings emphasize the need for targeted prevention and regulatory strategies to address the growing accessibility and appeal of e-cigarettes.

A lack of regulation, extensive advertising exposure, and widespread misinformation about e-cigarette safety compound the issue, underscoring the need for comprehensive research and regulation across Latin America. We recommend developing robust prevention programs that engage diverse stakeholders, integrate education, social media campaigns, and school-based policies, and implement regulatory measures such as age restrictions, online sales limits, and bans on youth-targeted flavors.

## Ethics approval and consent to participate

This study is based on publicly available data and, as a systematic review, is exempt from requiring ethical approval.

## Funding sources

This research did not receive any specific grant from funding agencies in the public, commercial, or not-for-profit sectors.

## CRediT authorship contribution statement

**Juan S. Izquierdo-Condoy:** Writing – review & editing, Writing – original draft, Visualization, Validation, Supervision, Software, Resources, Project administration, Methodology, Investigation, Formal analysis, Data curation, Conceptualization. **Kenny Ruiz Sosa:** Writing – original draft, Visualization, Resources, Methodology, Investigation, Formal analysis, Data curation. **Camila Salazar-Santoliva:** Writing – original draft, Visualization, Methodology, Investigation, Formal analysis, Data curation. **Natalia Restrepo:** Writing – original draft, Visualization, Resources, Methodology, Investigation, Data curation. **Guillermo Olaya-Villareal:** Writing – original draft, Visualization, Software, Resources, Methodology, Investigation, Data curation. **Juan S. Castillo-Concha:** Writing – original draft, Validation, Resources, Methodology, Investigation, Data curation. **Valentina Loaiza-Guevara:** Writing – original draft, Visualization, Validation, Resources, Methodology, Investigation, Data curation. **Esteban Ortiz-Prado:** Writing – review & editing, Validation, Supervision, Project administration, Investigation, Funding acquisition.

## Declaration of competing interest

The authors declare that they have no known competing financial interests or personal relationships that could have appeared to influence the work reported in this paper.

## Data Availability

No data was used for the research described in the article.

## Appendix A Supplementary data

Supplementary material Image 1
