# Supplementary material for: E-cigarette use among adolescents in Latin America: A systematic review of prevalence and associated factors
Source: Prev Med Rep. 2024 Dec 19;49:102952. doi: 10.1016/j.pmedr.2024.102952 (PMC11733054; doi:10.1016/j.pmedr.2024.102952)
Supplement: Supplementary file 1 — Supplementary material [file mmc1.docx]

**Supplementary Table 1:** Newcastle - Ottawa quality assessment scale.

| **Study** | **1** | **2** | **3** | **4** | **5** | **6** | **7** | **8** | **Score** |
| --- | --- | --- | --- | --- | --- | --- | --- | --- | --- |
| Morello P., et al., 2018 | * | * | * | * | ** | * | * | * | 9 |
| Barrientos-Gutierrez I., et al., 2019 | * | * | * | * | ** | * | * | * | 9 |
| Thrasher J., et al., 2016 | * | * | * |  | ** | * |  |  | 6 |
| Morello P., et al., 2016 | * | * | * | * | ** | * | * | * | 9 |

**Note:** A study can receive a maximum of one star for each item numbered within the Selection and Result categories. A maximum of two stars can be awarded for comparability.

**Selection**

1. Representativeness of the exposed court.

2. Selection of the unexposed court.

3. Exposure determination.

4. Demonstration that the current outcome of interest was not present at baseline.

**Comparability**

5. Cohort comparability based on design or analysis.

**Results**

6. Evaluation of the result.

7. Was the follow-up long enough for the results to occur?

8. Adequacy of cohort follow-up.

**Interpretation**

Good quality: 3 or 4 stars in the selection domain and 1 or 2 stars in the comparability domain and 2 or 3 stars in the outcome/exposure domain.
Acceptable quality: 2 stars in the selection domain and 1 or 2 stars in the comparability domain and 2 or 3 stars in the outcome/exposure domain.
Poor quality: 0 or 1 star in the selection domain or 0 stars in the comparability domain or 0 or 1 stars in the outcome/ exposure domain

**Supplementary Table 2:** JBI critical appraisal checklist for analytical cross-sectional studies

| **Study** | **1** | **2** | **3** | **4** | **5** | **6** | **7** | **8** | **Score** |
| --- | --- | --- | --- | --- | --- | --- | --- | --- | --- |
| Ocasio-Peña C., et al., 2023 | NO | NO | YES | YES | NO | NO | YES | YES | 4 |
| Barrera-Núñez D., et al., 2023 | YES | NO | YES | YES | NO | NO | YES | YES | 5 |
| Cortés E., et al., 2023 | YES | NO | YES | YES | NO | NO | YES | YES | 5 |
| Zavala-Arciniega L., et al., 2020 | YES | YES | YES | YES | YES | YES | YES | YES | 8 |
| Rodríguez-Bolaños R., et al., 2020 | YES | YES | YES | YES | YES | YES | YES | YES | 8 |
| Gottschlich A., et al., 2020 | YES | YES | YES | YES | YES | YES | YES | YES | 8 |
| Chérrez-Ojeda I., et al., 2024 | YES | YES | YES | YES | YES | YES | YES | YES | 8 |
| Isea S., et al., 2023 | NO | NO | YES | YES | YES | NO | NO | NO | 3 |
| Scoppetta O., and Villamil A., 2023 | YES | YES | NO | YES | NO | NO | NO | NO | 3 |
| Morello P., et al. 2020 | YES | YES | YES | YES | NO | NO | YES | YES | 6 |

1. Were the criteria for inclusion in the sample clearly defined?

2. Were the study subjects and setting described in detail?

3. Was the exposure measured in a valid and reliable way?

4. Were standard and objective criteria used to measure the condition?

5.Were confounding factors identified?

6. Were strategies established to deal with confounding factors?

7. Were the outcomes measured in a valid and reliable way?

8. Was an appropriate statistical analysis used?

**Interpretation**

Quality scores were categorized into three groups: Low: 1-4, Moderate: 5-7, and High: >7
